# [Di­aqua­sesqui(nitrato-κ*O*)hemi(perchlorato-κ*O*)copper(II)]-μ-{bis­[5-methyl-3-(pyridin-2-yl)-1*H*-pyrazol-4-yl] selenide}-[tri­aqua­(perchlorato-κ*O*)copper(II)] nitrate monohydrate

**DOI:** 10.1107/S1600536813012178

**Published:** 2013-05-15

**Authors:** Maksym Seredyuk, Vadim A. Pavlenko, Kateryna O. Znovjyak, Elzbieta Gumienna-Kontecka, Turganbay S. Iskenderov

**Affiliations:** a National Taras Shevchenko University, Department of Chemistry, Volodymyrska str. 64, 01601 Kyiv, Ukraine; b Faculty of Chemistry, University of Wroclaw, 14, F. Joliot-Curie Str., 50383, Wroclaw, Poland

## Abstract

In the binuclear title complex, [Cu_2_(ClO_4_)_1.5_(NO_3_)_1.5_(C_18_H_16_N_6_Se)(H_2_O)_5_]NO_3_·H_2_O, both Cu^II^ ions are hexa­coordinated by O and N atoms, thus forming axially elongated CuO_4_N_2_ octa­hedra. The equatorial plane of each octa­hedron is formed by one chelating pyrazole–pyridine fragment of the organic ligand and two water mol­ecules. The axial positions in one octa­hedron are occupied by a water mol­ecule and a monodentately coordinated perchlorate anion, while those in the other are occupied by a nitrate anion and a disordered perchlorate/nitrate anion with equal site occupancy. The pyrazole–pyridine units of the organic selenide are *trans*-oriented to each other with a C—Se—C angle of 96.01 (14)°. In the crystal, uncoordinated nitrate anions and the coordinating water mol­ecules are involved in O—H⋯O and N—H⋯O hydrogen bonds, forming a bridge between the pyrazole group and the coordinating water mol­ecules. Further O—H⋯O hydrogen bonds between the complex mol­ecules and a π–π stacking inter­action with a centroid–centroid distance of 3.834 (4) Å are also observed.

## Related literature

For structural studies of related pyrazolylselenides, see: Seredyuk *et al.* (2010 ▶) and for structural studies of *d*-metal complexes of bis­(3,5-dimethyl-1*H*-pyrazol-4-yl)selenide, see: Seredyuk *et al.* (2007 ▶). For related structures, see: Fritsky *et al.* (2004 ▶); Kanderal *et al.* (2005 ▶); Moroz *et al.* (2010 ▶).
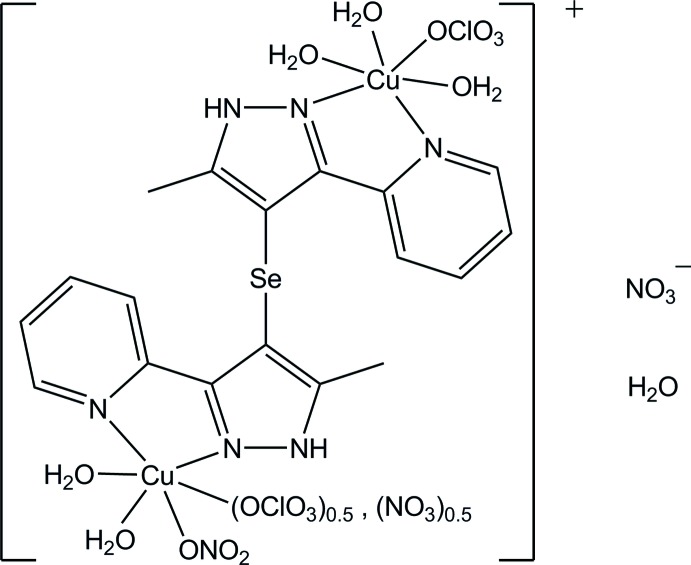


## Experimental

### 

#### Crystal data

[Cu_2_(ClO_4_)_1.5_(NO_3_)_1.5_(C_18_H_16_N_6_Se)(H_2_O)_5_]NO_3_·H_2_O
*M* *_r_* = 934.72
Triclinic, 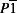

*a* = 9.7233 (6) Å
*b* = 13.1987 (7) Å
*c* = 13.3217 (8) Å
α = 93.510 (4)°
β = 108.858 (5)°
γ = 93.494 (4)°
*V* = 1608.93 (16) Å^3^
*Z* = 2
Mo *K*α radiation
μ = 2.67 mm^−1^
*T* = 100 K
0.30 × 0.25 × 0.12 mm

#### Data collection

Bruker SMART APEXII CCD diffractometer
Absorption correction: multi-scan (*SADABS*; Bruker, 2009 ▶) *T*_min_ = 0.468, *T*_max_ = 0.728
11167 measured reflections
7085 independent reflections
5817 reflections with *I* > 2σ(*I*)
*R*_int_ = 0.073

#### Refinement

*R*[*F* ^2^ > 2σ(*F* ^2^)] = 0.041
*wR*(*F* ^2^) = 0.113
*S* = 1.02
7085 reflections
498 parameters
12 restraints
H atoms treated by a mixture of independent and constrained refinement
Δρ_max_ = 1.00 e Å^−3^
Δρ_min_ = −0.95 e Å^−3^

### 

Data collection: *APEX2* (Bruker, 2009 ▶); cell refinement: *SAINT* (Bruker, 2009 ▶); data reduction: *SAINT*; program(s) used to solve structure: *SIR2004* (Burla *et al.*, 2005 ▶); program(s) used to refine structure: *SHELXL97* (Sheldrick, 2008 ▶); molecular graphics: *DIAMOND* (Brandenburg, 2009 ▶); software used to prepare material for publication: *SHELXL97*.

## Supplementary Material

Crystal structure: contains datablock(s) I, global. DOI: 10.1107/S1600536813012178/is5261sup1.cif

Structure factors: contains datablock(s) I. DOI: 10.1107/S1600536813012178/is5261Isup2.hkl

Supplementary material file. DOI: 10.1107/S1600536813012178/is5261Isup3.cdx

Additional supplementary materials:  crystallographic information; 3D view; checkCIF report

## Figures and Tables

**Table 1:** Hydrogen-bond geometry (Å, °)

*D*—H⋯*A*	*D*—H	H⋯*A*	*D*⋯*A*	*D*—H⋯*A*
O1—H2*O*1⋯O10	0.83 (2)	1.93 (2)	2.737 (3)	164 (4)
O1—H1*O*1⋯O18^i^	0.81 (2)	2.00 (2)	2.801 (6)	174 (5)
O1—H1*O*1⋯O20^i^	0.81 (2)	1.99 (3)	2.747 (6)	155 (4)
O2—H1*O*2⋯O13^ii^	0.83 (2)	1.85 (2)	2.660 (3)	163 (4)
O2—H2*O*2⋯O3^iii^	0.83 (2)	1.99 (2)	2.805 (4)	167 (4)
O3—H1*O*3⋯O11^i^	0.83 (2)	2.03 (2)	2.842 (4)	166 (4)
O3—H1*O*3⋯O12^i^	0.83 (2)	2.47 (3)	3.130 (4)	137 (4)
O4—H1*O*4⋯O10^iv^	0.83 (2)	1.94 (2)	2.762 (3)	174 (4)
O4—H2*O*4⋯O1*W* ^v^	0.84 (2)	1.88 (2)	2.717 (4)	174 (5)
O5—H1*O*5⋯O13	0.82 (2)	1.87 (3)	2.617 (4)	150 (4)
O5—H2*O*5⋯O16	0.84 (2)	1.97 (3)	2.719 (11)	148 (4)
O5—H2*O*5⋯O16	0.84 (2)	1.97 (3)	2.719 (11)	148 (4)
O5—H2*O*5⋯O22	0.84 (2)	2.07 (3)	2.784 (10)	142 (4)
O1*W*—H2*W*1⋯O6^iv^	0.83 (2)	2.10 (3)	2.800 (4)	142 (4)
O1*W*—H1*W*1⋯O16^vi^	0.84 (2)	2.12 (3)	2.833 (9)	144 (4)
O1*W*—H1*W*1⋯O22^vi^	0.84 (2)	2.23 (3)	2.977 (11)	149 (4)
N2—H1*N*2⋯O11	0.86	1.98	2.829 (4)	168
N5—H1*N*5⋯O1*W*	0.86	1.94	2.762 (4)	160

Symmetry codes: (i) 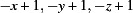; (ii) 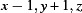; (iii) 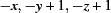; (iv) 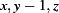; (v) 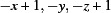; (vi) 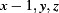.

**Fig. 1.**
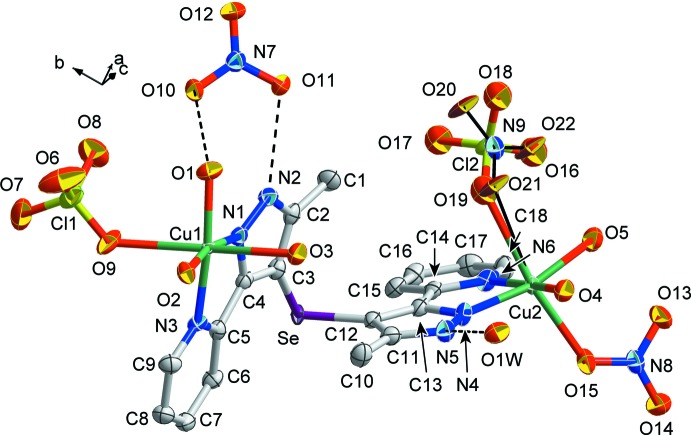
The asymmetric unit of the title compound, showing the labeling scheme and 90% probability displacement ellipsoids. Hydrogen bonds are indicated by dashed lines. H atoms are omitted for clarity.

**Fig. 2.**
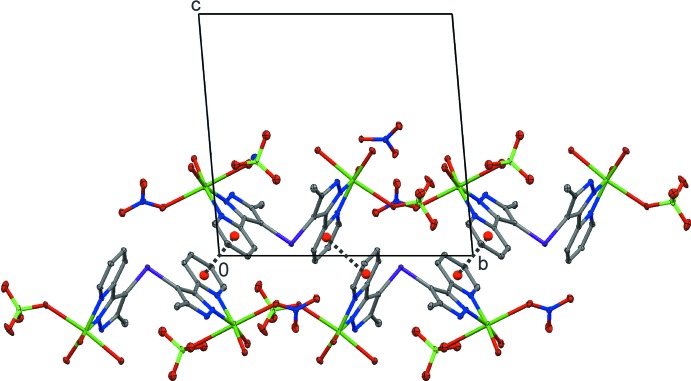
Projection of a fragment of the crystal packing along the a axis showing π–π stacking intractions between the pyridine groups (dashed lines).

## supplementary crystallographic information

### Comment

Pyrazole-derived ligands are widely used in molecular magnetism, bioinorganic modelling and supramolecular chemistry due to their bridging nature and possibility for easy functionalization. As a part of our synthetic and structural study of pyrazolylselenides (Seredyuk *et al.*, 2010), and their complexes with *d*-metals (Seredyuk *et al.*, 2007), we report here the molecular and crystal structures of the title compound (Fig. 1).

The title compound, [Cu_2_(H_2_O)_5_(NO_3_)_1.5_(ClO_4_)_1.5_(C_18_H_16_N_6_Se)]^+^.NO_3_^-^.H_2_O, is a binuclear complex formed by bis(3-methyl-5-(pyridin-2-yl)-1*H*-pyrazol-4-yl)selenide (Seredyuk *et al.*, 2010), where both Cu^II^ ions are surrounded by fuor O and two N donor atoms which form coordination polyhedra best described as axially elongated octahedra. In both, the equatorial planes are formed by the chelating pyrazole-pyridine fragment of the organic ligand [Cu—N 1.942 (3)–2.023 (3) Å] and two water molecules [Cu—O 1.948 (3)–1.972 (3) Å], whereas the axial positions are occupied by the water molecule [Cu—O 2.419 (3) Å] and the monodentately coordinated perchlorate anion [Cu1—O 2.489 (3) Å] or the perchlorate/nitrate anion [Cu2—O 2.643 (11)/2.688 (11) Å] and the nitrate anion [Cu2—O 2.445 (3) Å]. The organic selenide is trans-oriented with the angle C—Se—C equal to 96.01 (14)°. The C—N and C—C bond lengths in the pyridine rings are normal for 2-substituted pyridine derivatives (Fritsky *et al.*, 2004; Kanderal *et al.*, 2005; Moroz *et al.*, 2010).

An additional nitrate anion balancing the charge of the complex molecule serves a bridge being involved in intermolecular hydrogen bonds between the NH group of a pyrazole moiety [N···O = 2.829 (4) Å] and the water molecule coordinated to the Cu1 ion [O···O = 2.737 (3) Å]. The second NH group of the ligand molecule is bonded with the water molecule [N···O = 2.762 (3) Å]. Also, numerous intermolecular hydrogen bonds are observed between water molecules and perchlorate and nitrate anions with O···O distances in the range of 2.660 (3)–3.022 (5) Å. The distances centroid-centroid between the closest coplanar pyridine fragments of the neighboring molecules are equal to 3.834 (4) and 4.010 (4) Å (Fig. 2).

### Experimental

In a solution of Cu(NO_3_)_2_.6H_2_O (0.144 g, 0.468 mmol) and NaClO_4_ (0.122 g, 1 mmol) in 5 ml of water a batch of bis(3-methyl-5-(pyridin-2-yl)-1*H*-pyrazol-4-yl)selenide.MeOH (0.1 g, 0.234 mmol) (Seredyuk *et al.*, 2010) was dissolved. After several weeks well formed green crystals were formed and isolated. Analysis, calculated for C_18_H_28_Cl_1.5_Cu_2_N_8.5_O_19.5_Se: C 23.13, H 3.02, N 12.74%; found: C 23.17, H 3.04, N 12.01%.

### Refinement

The NH and water H atoms were located in a difference Fourier map. The positions of water H atoms were refined with distance restraint of O—H = 0.84 (2) Å, and with *U*_iso_(H) = 1.5*U*_eq_(O), but H atoms of the NH groups were constrained to ride on their parent atom, with N—H = 0.86 Å, and with *U*_iso_(H) = 1.2*U*_eq_(N). The C-bound H atoms were positioned geometrically and refined as riding atoms, with C—H = 0.93(CH), 0.96(CH_3_), and with *U*_iso_(H) = 1.2 or 1.5*U*_eq_(C) for CH and CH_3_, respectively. It was found that one of the coordinated perchlorate ions occupy almost the same location with the nitrate ion, both ions were modelled as disordered over two positions with site occupancies of 0.5. For the disordered perchlorate/nitrate anion, each set of four/three oxygen atoms was restrained to have the same anisotropic displacement parameters.

### Crystal data

**Table:**

[Cu_2_(ClO_4_)_1.5_(NO_3_)_1.5_(C_18_H_16_N_6_Se)(H_2_O)_5_]NO_3_·H_2_O	*Z* = 2
*M**_r_* = 934.72	*F*(000) = 938
Triclinic, *P*1	*D*_x_ = 1.929 Mg m^−^^3^
Hall symbol: -P 1	Mo *K*α radiation, λ = 0.71073 Å
*a* = 9.7233 (6) Å	Cell parameters from 6705 reflections
*b* = 13.1987 (7) Å	θ = 2.6–30.3°
*c* = 13.3217 (8) Å	µ = 2.67 mm^−^^1^
α = 93.510 (4)°	*T* = 100 K
β = 108.858 (5)°	Block, green
γ = 93.494 (4)°	0.30 × 0.25 × 0.12 mm
*V* = 1608.93 (16) Å^3^	

### Data collection

**Table:**

Bruker SMART APEXII CCD diffractometer	7085 independent reflections
Radiation source: fine-focus sealed tube	5817 reflections with *I* > 2σ(*I*)
Flat graphite crystal monochromator	*R*_int_ = 0.073
Detector resolution: 16 pixels mm^-1^	θ_max_ = 28.4°, θ_min_ = 3.5°
φ and ω scans	*h* = −10→12
Absorption correction: multi-scan (*SADABS*; Bruker, 2009)	*k* = −17→17
*T*_min_ = 0.468, *T*_max_ = 0.728	*l* = −17→16
11167 measured reflections	

### Refinement

**Table:**

Refinement on *F*^2^	Primary atom site location: structure-invariant direct methods
Least-squares matrix: full	Secondary atom site location: difference Fourier map
*R*[*F*^2^ > 2σ(*F*^2^)] = 0.041	Hydrogen site location: inferred from neighbouring sites
*wR*(*F*^2^) = 0.113	H atoms treated by a mixture of independent and constrained refinement
*S* = 1.02	*w* = 1/[σ^2^(*F*_o_^2^) + (0.0615*P*)^2^] where *P* = (*F*_o_^2^ + 2*F*_c_^2^)/3
7085 reflections	(Δ/σ)_max_ = 0.001
498 parameters	Δρ_max_ = 1.00 e Å^−^^3^
12 restraints	Δρ_min_ = −0.95 e Å^−^^3^

### Special details

**Table:**

Geometry. All esds (except the esd in the dihedral angle between two l.s. planes) are estimated using the full covariance matrix. The cell esds are taken into account individually in the estimation of esds in distances, angles and torsion angles; correlations between esds in cell parameters are only used when they are defined by crystal symmetry. An approximate (isotropic) treatment of cell esds is used for estimating esds involving l.s. planes.
Refinement. Refinement of F^2^ against ALL reflections. The weighted R-factor wR and goodness of fit S are based on F^2^, conventional R-factors R are based on F, with F set to zero for negative F^2^. The threshold expression of F^2^ > 2sigma(F^2^) is used only for calculating R-factors(gt) etc. and is not relevant to the choice of reflections for refinement. R-factors based on F^2^ are statistically about twice as large as those based on F, and R- factors based on ALL data will be even larger.

### Fractional atomic coordinates and isotropic or equivalent isotropic displacement parameters (Å^2^)

**Table:**

	*x*	*y*	*z*	*U*_iso_*/*U*_eq_	Occ. (<1)
Se	0.30579 (4)	0.28810 (2)	0.04893 (3)	0.01672 (10)	
Cu1	0.07983 (4)	0.55346 (3)	0.31262 (3)	0.01379 (10)	
Cu2	0.60531 (4)	−0.03338 (3)	0.28618 (3)	0.01818 (11)	
Cl1	0.08419 (9)	0.80696 (6)	0.22351 (8)	0.02334 (19)	
O1	0.2168 (3)	0.63956 (19)	0.4340 (2)	0.0209 (5)	
H2O1	0.284 (4)	0.669 (3)	0.419 (4)	0.031*	
H1O1	0.173 (4)	0.680 (3)	0.457 (3)	0.031*	
O2	−0.0895 (2)	0.57907 (18)	0.35723 (19)	0.0166 (5)	
H1O2	−0.137 (4)	0.626 (2)	0.329 (3)	0.025*	
H2O2	−0.079 (4)	0.577 (3)	0.4215 (17)	0.025*	
O1W	0.2383 (3)	0.01642 (19)	0.4205 (2)	0.0228 (5)	
H1W1	0.181 (4)	0.059 (3)	0.428 (4)	0.034*	
H2W1	0.216 (5)	−0.0443 (17)	0.397 (4)	0.034*	
O3	0.1043 (3)	0.42346 (18)	0.4361 (2)	0.0192 (5)	
H1O3	0.189 (3)	0.406 (3)	0.457 (3)	0.029*	
H2O3	0.056 (4)	0.370 (2)	0.410 (3)	0.029*	
O4	0.5283 (3)	−0.06721 (19)	0.4011 (2)	0.0214 (5)	
H1O4	0.505 (5)	−0.1278 (17)	0.405 (4)	0.032*	
H2O4	0.596 (4)	−0.049 (3)	0.458 (2)	0.032*	
O5	0.7923 (3)	−0.08153 (19)	0.3647 (2)	0.0245 (6)	
H1O5	0.802 (5)	−0.139 (2)	0.341 (4)	0.037*	
H2O5	0.865 (3)	−0.045 (3)	0.362 (4)	0.037*	
O6	0.0385 (3)	0.8548 (2)	0.3056 (3)	0.0499 (10)	
O7	0.0311 (3)	0.8566 (2)	0.1280 (3)	0.0410 (8)	
O8	0.2401 (3)	0.8125 (2)	0.2577 (2)	0.0368 (7)	
O9	0.0257 (3)	0.70178 (18)	0.2037 (2)	0.0287 (6)	
O10	0.4717 (3)	0.72896 (18)	0.4218 (2)	0.0255 (6)	
O11	0.5918 (3)	0.59929 (17)	0.4819 (2)	0.0218 (5)	
O12	0.6898 (3)	0.75338 (19)	0.5375 (2)	0.0299 (6)	
O13	0.7312 (3)	−0.2750 (2)	0.2985 (3)	0.0379 (7)	
O14	0.5604 (3)	−0.36909 (19)	0.1776 (2)	0.0290 (6)	
O15	0.5431 (3)	−0.20641 (19)	0.1993 (2)	0.0249 (5)	
N1	0.2334 (3)	0.4991 (2)	0.2624 (2)	0.0149 (5)	
N2	0.3773 (3)	0.4906 (2)	0.3050 (2)	0.0159 (5)	
H1N2	0.4325	0.5242	0.3628	0.019*	
N3	−0.0450 (3)	0.47889 (19)	0.1744 (2)	0.0145 (5)	
N4	0.4412 (3)	0.0459 (2)	0.2249 (2)	0.0169 (6)	
N5	0.3236 (3)	0.0687 (2)	0.2505 (2)	0.0179 (6)	
H1N5	0.2936	0.0381	0.2958	0.021*	
N6	0.6606 (3)	0.0214 (2)	0.1646 (2)	0.0191 (6)	
N7	0.5859 (3)	0.6952 (2)	0.4812 (2)	0.0188 (6)	
N8	0.6108 (3)	−0.2844 (2)	0.2239 (2)	0.0188 (6)	
C1	0.5770 (3)	0.3955 (3)	0.2785 (3)	0.0219 (7)	
H1A	0.6415	0.4534	0.3163	0.033*	
H1B	0.6008	0.3744	0.2163	0.033*	
H1C	0.5877	0.3407	0.3239	0.033*	
C2	0.4246 (4)	0.4232 (2)	0.2463 (3)	0.0161 (6)	
C3	0.3034 (3)	0.3858 (2)	0.1598 (3)	0.0143 (6)	
C4	0.1865 (3)	0.4339 (2)	0.1747 (3)	0.0140 (6)	
C5	0.0275 (3)	0.4270 (2)	0.1190 (3)	0.0140 (6)	
C6	−0.0450 (4)	0.3737 (2)	0.0211 (3)	0.0171 (6)	
H6	0.0063	0.3376	−0.0152	0.021*	
C7	−0.1951 (4)	0.3752 (2)	−0.0219 (3)	0.0187 (7)	
H7	−0.2458	0.3391	−0.0869	0.022*	
C8	−0.2691 (4)	0.4306 (3)	0.0323 (3)	0.0188 (7)	
H8	−0.3695	0.4336	0.0038	0.023*	
C9	−0.1901 (3)	0.4817 (2)	0.1305 (3)	0.0177 (7)	
H9	−0.2395	0.5192	0.1672	0.021*	
C10	0.1243 (4)	0.1828 (3)	0.2098 (3)	0.0210 (7)	
H10A	0.0756	0.1312	0.2370	0.031*	
H10B	0.0607	0.1990	0.1420	0.031*	
H10C	0.1498	0.2428	0.2586	0.031*	
C11	0.2582 (3)	0.1449 (2)	0.1969 (3)	0.0170 (6)	
C12	0.3424 (3)	0.1760 (2)	0.1345 (3)	0.0151 (6)	
C13	0.4547 (4)	0.1103 (2)	0.1543 (3)	0.0161 (6)	
C14	0.5762 (3)	0.0938 (2)	0.1147 (3)	0.0169 (6)	
C15	0.6019 (4)	0.1410 (2)	0.0314 (3)	0.0180 (7)	
H15	0.5442	0.1914	−0.0008	0.022*	
C16	0.7149 (4)	0.1119 (3)	−0.0031 (3)	0.0205 (7)	
H16	0.7333	0.1419	−0.0594	0.025*	
C17	0.8007 (4)	0.0369 (3)	0.0477 (3)	0.0228 (7)	
H17	0.8768	0.0159	0.0257	0.027*	
C18	0.7700 (4)	−0.0058 (3)	0.1317 (3)	0.0219 (7)	
H18	0.8278	−0.0550	0.1662	0.026*	
Cl2	0.8810 (3)	0.1792 (2)	0.3891 (2)	0.0147 (5)	0.50
O16	0.9609 (11)	0.0934 (7)	0.3751 (9)	0.0270 (7)	0.50
O17	0.8847 (5)	0.2502 (4)	0.3146 (5)	0.0270 (7)	0.50
O18	0.9531 (6)	0.2249 (4)	0.4990 (5)	0.0270 (7)	0.50
O19	0.7361 (11)	0.1453 (8)	0.3799 (7)	0.0270 (7)	0.50
N9	0.8701 (15)	0.1712 (11)	0.4062 (11)	0.0188 (6)	0.50
O20	0.9164 (6)	0.2618 (4)	0.4398 (6)	0.0363 (11)	0.50
O21	0.7413 (12)	0.1391 (8)	0.4031 (8)	0.0363 (11)	0.50
O22	0.9545 (12)	0.1050 (7)	0.3892 (10)	0.0363 (11)	0.50

### Atomic displacement parameters (Å^2^)

**Table:**

	*U*^11^	*U*^22^	*U*^33^	*U*^12^	*U*^13^	*U*^23^
Se	0.02294 (18)	0.01469 (16)	0.01541 (17)	0.00675 (12)	0.00905 (13)	0.00297 (12)
Cu1	0.01339 (19)	0.01215 (19)	0.0161 (2)	0.00040 (14)	0.00579 (15)	−0.00181 (15)
Cu2	0.0180 (2)	0.0160 (2)	0.0223 (2)	0.00449 (15)	0.00766 (17)	0.00644 (17)
Cl1	0.0206 (4)	0.0162 (4)	0.0349 (5)	0.0012 (3)	0.0124 (4)	−0.0028 (3)
O1	0.0184 (12)	0.0196 (12)	0.0252 (13)	−0.0029 (10)	0.0102 (10)	−0.0060 (10)
O2	0.0161 (11)	0.0188 (11)	0.0161 (12)	0.0067 (9)	0.0059 (9)	0.0029 (10)
O1W	0.0244 (13)	0.0199 (12)	0.0251 (14)	−0.0013 (10)	0.0110 (11)	−0.0021 (11)
O3	0.0206 (12)	0.0156 (11)	0.0223 (13)	0.0012 (9)	0.0080 (10)	0.0033 (10)
O4	0.0241 (13)	0.0173 (12)	0.0221 (13)	−0.0002 (10)	0.0065 (10)	0.0041 (10)
O5	0.0217 (13)	0.0182 (12)	0.0334 (15)	0.0038 (10)	0.0080 (11)	0.0050 (11)
O6	0.0407 (18)	0.0424 (18)	0.072 (2)	−0.0143 (14)	0.0361 (17)	−0.0361 (18)
O7	0.0314 (16)	0.0275 (14)	0.064 (2)	0.0057 (12)	0.0119 (15)	0.0213 (15)
O8	0.0202 (14)	0.0473 (18)	0.0435 (18)	0.0056 (12)	0.0102 (12)	0.0067 (14)
O9	0.0479 (17)	0.0127 (12)	0.0239 (14)	−0.0045 (11)	0.0119 (12)	−0.0032 (10)
O10	0.0159 (12)	0.0209 (12)	0.0356 (16)	0.0022 (10)	0.0013 (11)	0.0113 (11)
O11	0.0239 (13)	0.0132 (11)	0.0247 (13)	0.0002 (9)	0.0030 (10)	0.0025 (10)
O12	0.0255 (14)	0.0192 (12)	0.0347 (16)	−0.0033 (10)	−0.0023 (11)	−0.0044 (11)
O13	0.0268 (15)	0.0255 (14)	0.0448 (18)	0.0119 (12)	−0.0116 (13)	−0.0029 (13)
O14	0.0273 (14)	0.0223 (13)	0.0324 (15)	0.0000 (11)	0.0039 (11)	−0.0017 (12)
O15	0.0233 (13)	0.0240 (13)	0.0262 (14)	0.0090 (10)	0.0041 (10)	0.0082 (11)
N1	0.0136 (13)	0.0120 (12)	0.0200 (14)	−0.0001 (10)	0.0065 (11)	0.0019 (11)
N2	0.0143 (13)	0.0152 (13)	0.0174 (14)	0.0002 (10)	0.0049 (11)	−0.0002 (11)
N3	0.0151 (13)	0.0113 (12)	0.0180 (14)	−0.0006 (10)	0.0076 (11)	−0.0014 (11)
N4	0.0208 (14)	0.0136 (13)	0.0194 (15)	0.0049 (11)	0.0094 (11)	0.0055 (11)
N5	0.0164 (13)	0.0190 (14)	0.0212 (15)	0.0012 (11)	0.0092 (11)	0.0067 (12)
N6	0.0207 (14)	0.0165 (13)	0.0205 (15)	0.0020 (11)	0.0071 (12)	0.0027 (12)
N7	0.0177 (14)	0.0167 (13)	0.0227 (15)	0.0008 (11)	0.0073 (12)	0.0036 (12)
N8	0.0166 (13)	0.0199 (14)	0.0211 (15)	0.0055 (11)	0.0067 (11)	0.0057 (11)
C1	0.0149 (16)	0.0207 (16)	0.031 (2)	0.0023 (13)	0.0085 (14)	0.0022 (15)
C2	0.0193 (16)	0.0127 (14)	0.0189 (16)	0.0009 (12)	0.0094 (13)	0.0047 (13)
C3	0.0168 (15)	0.0129 (14)	0.0156 (15)	0.0032 (12)	0.0082 (12)	0.0011 (12)
C4	0.0180 (15)	0.0105 (14)	0.0139 (15)	−0.0005 (12)	0.0060 (12)	0.0021 (12)
C5	0.0149 (15)	0.0123 (14)	0.0158 (16)	0.0012 (11)	0.0059 (12)	0.0028 (12)
C6	0.0209 (16)	0.0139 (15)	0.0168 (16)	0.0014 (12)	0.0072 (13)	−0.0030 (13)
C7	0.0225 (17)	0.0141 (15)	0.0161 (16)	−0.0009 (13)	0.0022 (13)	0.0007 (13)
C8	0.0157 (15)	0.0194 (16)	0.0186 (17)	−0.0006 (13)	0.0025 (13)	0.0011 (13)
C9	0.0171 (16)	0.0185 (16)	0.0174 (17)	−0.0007 (13)	0.0064 (13)	0.0002 (13)
C10	0.0188 (16)	0.0198 (16)	0.0272 (19)	0.0021 (13)	0.0106 (14)	0.0051 (14)
C11	0.0155 (15)	0.0162 (15)	0.0179 (16)	−0.0015 (12)	0.0043 (13)	−0.0007 (13)
C12	0.0180 (15)	0.0111 (14)	0.0146 (16)	−0.0002 (12)	0.0035 (12)	0.0009 (12)
C13	0.0201 (16)	0.0120 (14)	0.0161 (16)	0.0001 (12)	0.0057 (13)	0.0018 (12)
C14	0.0172 (15)	0.0104 (14)	0.0215 (17)	0.0004 (12)	0.0048 (13)	−0.0021 (13)
C15	0.0203 (16)	0.0126 (14)	0.0209 (17)	−0.0006 (12)	0.0069 (13)	0.0000 (13)
C16	0.0266 (18)	0.0179 (16)	0.0193 (17)	0.0003 (13)	0.0111 (14)	0.0015 (14)
C17	0.0197 (17)	0.0241 (18)	0.0263 (19)	0.0003 (14)	0.0115 (14)	−0.0037 (15)
C18	0.0218 (17)	0.0196 (16)	0.0250 (19)	0.0065 (14)	0.0079 (14)	0.0023 (14)
Cl2	0.0151 (9)	0.0085 (8)	0.0224 (13)	−0.0033 (6)	0.0107 (7)	−0.0058 (8)
O16	0.0208 (15)	0.0273 (16)	0.0354 (19)	0.0073 (12)	0.0117 (13)	0.0038 (14)
O17	0.0208 (15)	0.0273 (16)	0.0354 (19)	0.0073 (12)	0.0117 (13)	0.0038 (14)
O18	0.0208 (15)	0.0273 (16)	0.0354 (19)	0.0073 (12)	0.0117 (13)	0.0038 (14)
O19	0.0208 (15)	0.0273 (16)	0.0354 (19)	0.0073 (12)	0.0117 (13)	0.0038 (14)
N9	0.0166 (13)	0.0199 (14)	0.0211 (15)	0.0055 (11)	0.0067 (11)	0.0057 (11)
O20	0.034 (2)	0.0206 (19)	0.061 (3)	−0.0118 (16)	0.030 (2)	−0.0110 (19)
O21	0.034 (2)	0.0206 (19)	0.061 (3)	−0.0118 (16)	0.030 (2)	−0.0110 (19)
O22	0.034 (2)	0.0206 (19)	0.061 (3)	−0.0118 (16)	0.030 (2)	−0.0110 (19)

### Geometric parameters (Å, º)

**Table:**

Se—C12	1.904 (3)	N4—N5	1.340 (4)
Se—C3	1.907 (3)	N5—C11	1.341 (4)
Cu1—O2	1.963 (2)	N5—H1N5	0.8600
Cu1—O1	1.969 (3)	N6—C18	1.334 (4)
Cu1—N1	1.978 (3)	N6—C14	1.360 (4)
Cu1—N3	2.005 (3)	C1—C2	1.479 (4)
Cu1—O3	2.419 (3)	C1—H1A	0.9600
Cu1—O9	2.489 (3)	C1—H1B	0.9600
Cu2—N4	1.942 (3)	C1—H1C	0.9600
Cu2—O5	1.948 (3)	C2—C3	1.394 (5)
Cu2—O4	1.972 (3)	C3—C4	1.398 (4)
Cu2—N6	2.023 (3)	C4—C5	1.477 (4)
Cu2—O15	2.445 (3)	C5—C6	1.388 (4)
Cu2—O19	2.643 (11)	C6—C7	1.387 (5)
Cl1—O7	1.426 (3)	C6—H6	0.9300
Cl1—O8	1.431 (3)	C7—C8	1.382 (5)
Cl1—O6	1.434 (3)	C7—H7	0.9300
Cl1—O9	1.443 (3)	C8—C9	1.391 (5)
O1—H2O1	0.826 (19)	C8—H8	0.9300
O1—H1O1	0.805 (19)	C9—H9	0.9300
O2—H1O2	0.834 (19)	C10—C11	1.479 (4)
O2—H2O2	0.831 (19)	C10—H10A	0.9600
O1W—H1W1	0.836 (19)	C10—H10B	0.9600
O1W—H2W1	0.832 (19)	C10—H10C	0.9600
O3—H1O3	0.831 (19)	C11—C12	1.403 (5)
O3—H2O3	0.818 (19)	C12—C13	1.404 (5)
O4—H1O4	0.829 (19)	C13—C14	1.464 (5)
O4—H2O4	0.836 (19)	C14—C15	1.388 (5)
O5—H1O5	0.823 (19)	C15—C16	1.386 (5)
O5—H2O5	0.842 (19)	C15—H15	0.9300
O10—N7	1.262 (4)	C16—C17	1.397 (5)
O11—N7	1.271 (3)	C16—H16	0.9300
O12—N7	1.232 (4)	C17—C18	1.389 (5)
O13—N8	1.260 (4)	C17—H17	0.9300
O14—N8	1.234 (4)	C18—H18	0.9300
O15—N8	1.259 (4)	Cl2—O17	1.413 (6)
N1—C4	1.340 (4)	Cl2—O19	1.415 (11)
N1—N2	1.344 (4)	Cl2—O16	1.447 (8)
N2—C2	1.346 (4)	Cl2—O18	1.475 (7)
N2—H1N2	0.8600	N9—O20	1.255 (15)
N3—C9	1.346 (4)	N9—O21	1.285 (17)
N3—C5	1.357 (4)	N9—O22	1.293 (15)
N4—C13	1.337 (4)		
			
C12—Se—C3	96.01 (14)	O10—N7—O11	118.5 (3)
O2—Cu1—O1	93.43 (10)	O14—N8—O15	121.3 (3)
O2—Cu1—N1	168.52 (10)	O14—N8—O13	120.1 (3)
O1—Cu1—N1	94.85 (11)	O15—N8—O13	118.6 (3)
O2—Cu1—N3	92.57 (11)	C2—C1—H1A	109.5
O1—Cu1—N3	170.62 (11)	C2—C1—H1B	109.5
N1—Cu1—N3	80.28 (11)	H1A—C1—H1B	109.5
O2—Cu1—O3	81.67 (9)	C2—C1—H1C	109.5
O1—Cu1—O3	85.52 (10)	H1A—C1—H1C	109.5
N1—Cu1—O3	91.06 (10)	H1B—C1—H1C	109.5
N3—Cu1—O3	102.50 (10)	N2—C2—C3	106.6 (3)
O2—Cu1—O9	88.12 (10)	N2—C2—C1	122.8 (3)
O1—Cu1—O9	91.62 (10)	C3—C2—C1	130.5 (3)
N1—Cu1—O9	99.57 (11)	C2—C3—C4	105.2 (3)
N3—Cu1—O9	81.37 (10)	C2—C3—Se	125.1 (2)
O3—Cu1—O9	169.20 (9)	C4—C3—Se	129.7 (2)
N4—Cu2—O5	166.31 (12)	N1—C4—C3	110.2 (3)
N4—Cu2—O4	90.67 (11)	N1—C4—C5	114.8 (3)
O5—Cu2—O4	90.21 (11)	C3—C4—C5	135.0 (3)
N4—Cu2—N6	79.71 (12)	N3—C5—C6	121.7 (3)
O5—Cu2—N6	98.40 (12)	N3—C5—C4	112.7 (3)
O4—Cu2—N6	169.88 (11)	C6—C5—C4	125.6 (3)
N4—Cu2—O15	107.91 (11)	C7—C6—C5	119.0 (3)
O5—Cu2—O15	85.70 (10)	C7—C6—H6	120.5
O4—Cu2—O15	92.59 (10)	C5—C6—H6	120.5
N6—Cu2—O15	93.36 (11)	C8—C7—C6	119.7 (3)
N4—Cu2—O19	83.5 (2)	C8—C7—H7	120.2
O5—Cu2—O19	82.9 (2)	C6—C7—H7	120.2
O4—Cu2—O19	95.1 (2)	C7—C8—C9	118.5 (3)
N6—Cu2—O19	80.7 (2)	C7—C8—H8	120.7
O15—Cu2—O19	166.2 (2)	C9—C8—H8	120.7
O7—Cl1—O8	109.63 (18)	N3—C9—C8	122.4 (3)
O7—Cl1—O6	110.2 (2)	N3—C9—H9	118.8
O8—Cl1—O6	109.33 (19)	C8—C9—H9	118.8
O7—Cl1—O9	109.16 (18)	C11—C10—H10A	109.5
O8—Cl1—O9	109.82 (18)	C11—C10—H10B	109.5
O6—Cl1—O9	108.64 (17)	H10A—C10—H10B	109.5
Cu1—O1—H2O1	113 (3)	C11—C10—H10C	109.5
Cu1—O1—H1O1	110 (3)	H10A—C10—H10C	109.5
H2O1—O1—H1O1	110 (4)	H10B—C10—H10C	109.5
Cu1—O2—H1O2	115 (3)	N5—C11—C12	106.6 (3)
Cu1—O2—H2O2	117 (3)	N5—C11—C10	122.1 (3)
H1O2—O2—H2O2	114 (4)	C12—C11—C10	131.3 (3)
H1W1—O1W—H2W1	127 (4)	C11—C12—C13	104.9 (3)
Cu1—O3—H1O3	111 (3)	C11—C12—Se	124.4 (2)
Cu1—O3—H2O3	114 (3)	C13—C12—Se	130.6 (3)
H1O3—O3—H2O3	103 (4)	N4—C13—C12	109.7 (3)
Cu2—O4—H1O4	118 (3)	N4—C13—C14	114.3 (3)
Cu2—O4—H2O4	105 (3)	C12—C13—C14	136.0 (3)
H1O4—O4—H2O4	106 (4)	N6—C14—C15	121.8 (3)
Cu2—O5—H1O5	112 (3)	N6—C14—C13	112.7 (3)
Cu2—O5—H2O5	114 (3)	C15—C14—C13	125.3 (3)
H1O5—O5—H2O5	105 (4)	C16—C15—C14	119.0 (3)
Cl1—O9—Cu1	132.23 (16)	C16—C15—H15	120.5
N8—O15—Cu2	128.4 (2)	C14—C15—H15	120.5
C4—N1—N2	106.2 (3)	C15—C16—C17	119.1 (3)
C4—N1—Cu1	115.8 (2)	C15—C16—H16	120.4
N2—N1—Cu1	135.9 (2)	C17—C16—H16	120.4
N1—N2—C2	111.8 (3)	C18—C17—C16	118.7 (3)
N1—N2—H1N2	124.1	C18—C17—H17	120.7
C2—N2—H1N2	124.1	C16—C17—H17	120.7
C9—N3—C5	118.7 (3)	N6—C18—C17	122.4 (3)
C9—N3—Cu1	125.6 (2)	N6—C18—H18	118.8
C5—N3—Cu1	115.6 (2)	C17—C18—H18	118.8
C13—N4—N5	106.9 (3)	O17—Cl2—O19	111.6 (4)
C13—N4—Cu2	116.9 (2)	O17—Cl2—O16	109.9 (5)
N5—N4—Cu2	134.7 (2)	O19—Cl2—O16	110.3 (6)
N4—N5—C11	111.7 (3)	O17—Cl2—O18	110.8 (4)
N4—N5—H1N5	124.1	O19—Cl2—O18	107.5 (5)
C11—N5—H1N5	124.1	O16—Cl2—O18	106.6 (5)
C18—N6—C14	119.0 (3)	Cl2—O19—Cu2	123.9 (6)
C18—N6—Cu2	126.5 (2)	O20—N9—O21	119.1 (12)
C14—N6—Cu2	114.5 (2)	O20—N9—O22	122.0 (12)
O12—N7—O10	121.2 (3)	O21—N9—O22	118.2 (12)
O12—N7—O11	120.3 (3)		
			
O7—Cl1—O9—Cu1	171.1 (2)	C12—Se—C3—C4	−109.6 (3)
O8—Cl1—O9—Cu1	50.8 (3)	N2—N1—C4—C3	−1.9 (3)
O6—Cl1—O9—Cu1	−68.7 (3)	Cu1—N1—C4—C3	−168.2 (2)
O2—Cu1—O9—Cl1	97.4 (2)	N2—N1—C4—C5	176.5 (2)
O1—Cu1—O9—Cl1	4.0 (2)	Cu1—N1—C4—C5	10.2 (3)
N1—Cu1—O9—Cl1	−91.2 (2)	C2—C3—C4—N1	2.1 (4)
N3—Cu1—O9—Cl1	−169.7 (2)	Se—C3—C4—N1	−178.8 (2)
O3—Cu1—O9—Cl1	78.5 (5)	C2—C3—C4—C5	−175.9 (3)
N4—Cu2—O15—N8	−170.6 (3)	Se—C3—C4—C5	3.2 (6)
O5—Cu2—O15—N8	10.9 (3)	C9—N3—C5—C6	−2.7 (5)
O4—Cu2—O15—N8	−79.1 (3)	Cu1—N3—C5—C6	−178.7 (2)
N6—Cu2—O15—N8	109.1 (3)	C9—N3—C5—C4	177.7 (3)
O19—Cu2—O15—N8	45.0 (8)	Cu1—N3—C5—C4	1.7 (3)
O2—Cu1—N1—C4	44.8 (7)	N1—C4—C5—N3	−7.7 (4)
O1—Cu1—N1—C4	−179.2 (2)	C3—C4—C5—N3	170.2 (3)
N3—Cu1—N1—C4	−7.3 (2)	N1—C4—C5—C6	172.7 (3)
O3—Cu1—N1—C4	95.2 (2)	C3—C4—C5—C6	−9.4 (6)
O9—Cu1—N1—C4	−86.7 (2)	N3—C5—C6—C7	1.1 (5)
O2—Cu1—N1—N2	−116.1 (5)	C4—C5—C6—C7	−179.3 (3)
O1—Cu1—N1—N2	20.0 (3)	C5—C6—C7—C8	1.0 (5)
N3—Cu1—N1—N2	−168.1 (3)	C6—C7—C8—C9	−1.5 (5)
O3—Cu1—N1—N2	−65.6 (3)	C5—N3—C9—C8	2.2 (5)
O9—Cu1—N1—N2	112.4 (3)	Cu1—N3—C9—C8	177.8 (2)
C4—N1—N2—C2	1.0 (3)	C7—C8—C9—N3	−0.1 (5)
Cu1—N1—N2—C2	163.1 (2)	N4—N5—C11—C12	−2.0 (4)
O2—Cu1—N3—C9	16.1 (3)	N4—N5—C11—C10	178.8 (3)
N1—Cu1—N3—C9	−172.9 (3)	N5—C11—C12—C13	1.9 (4)
O3—Cu1—N3—C9	98.2 (3)	C10—C11—C12—C13	−179.0 (3)
O9—Cu1—N3—C9	−71.6 (3)	N5—C11—C12—Se	−175.9 (2)
O2—Cu1—N3—C5	−168.2 (2)	C10—C11—C12—Se	3.2 (5)
N1—Cu1—N3—C5	2.8 (2)	C3—Se—C12—C11	53.5 (3)
O3—Cu1—N3—C5	−86.1 (2)	C3—Se—C12—C13	−123.7 (3)
O9—Cu1—N3—C5	104.1 (2)	N5—N4—C13—C12	0.1 (4)
O5—Cu2—N4—C13	70.2 (6)	Cu2—N4—C13—C12	−167.9 (2)
O4—Cu2—N4—C13	163.9 (3)	N5—N4—C13—C14	−178.1 (3)
N6—Cu2—N4—C13	−13.0 (2)	Cu2—N4—C13—C14	14.0 (4)
O15—Cu2—N4—C13	−103.2 (2)	C11—C12—C13—N4	−1.2 (4)
O19—Cu2—N4—C13	68.8 (3)	Se—C12—C13—N4	176.4 (2)
O5—Cu2—N4—N5	−93.4 (6)	C11—C12—C13—C14	176.4 (4)
O4—Cu2—N4—N5	0.2 (3)	Se—C12—C13—C14	−6.0 (6)
N6—Cu2—N4—N5	−176.6 (3)	C18—N6—C14—C15	−0.7 (5)
O15—Cu2—N4—N5	93.1 (3)	Cu2—N6—C14—C15	178.4 (2)
O19—Cu2—N4—N5	−94.9 (4)	C18—N6—C14—C13	175.9 (3)
C13—N4—N5—C11	1.2 (4)	Cu2—N6—C14—C13	−5.0 (4)
Cu2—N4—N5—C11	166.0 (2)	N4—C13—C14—N6	−5.4 (4)
N4—Cu2—N6—C18	−171.4 (3)	C12—C13—C14—N6	177.1 (4)
O5—Cu2—N6—C18	22.3 (3)	N4—C13—C14—C15	171.1 (3)
O4—Cu2—N6—C18	170.3 (5)	C12—C13—C14—C15	−6.5 (6)
O15—Cu2—N6—C18	−63.8 (3)	N6—C14—C15—C16	1.3 (5)
O19—Cu2—N6—C18	103.6 (4)	C13—C14—C15—C16	−174.9 (3)
N4—Cu2—N6—C14	9.6 (2)	C14—C15—C16—C17	−0.8 (5)
O5—Cu2—N6—C14	−156.7 (2)	C15—C16—C17—C18	−0.3 (5)
O4—Cu2—N6—C14	−8.8 (8)	C14—N6—C18—C17	−0.4 (5)
O15—Cu2—N6—C14	117.2 (2)	Cu2—N6—C18—C17	−179.4 (3)
O19—Cu2—N6—C14	−75.4 (3)	C16—C17—C18—N6	0.9 (5)
Cu2—O15—N8—O14	174.3 (2)	O17—Cl2—O19—Cu2	102.8 (5)
Cu2—O15—N8—O13	−4.6 (5)	O16—Cl2—O19—Cu2	−19.7 (8)
N1—N2—C2—C3	0.2 (4)	O18—Cl2—O19—Cu2	−135.5 (4)
N1—N2—C2—C1	−176.7 (3)	N4—Cu2—O19—Cl2	−136.8 (5)
N2—C2—C3—C4	−1.4 (3)	O5—Cu2—O19—Cl2	43.5 (5)
C1—C2—C3—C4	175.2 (3)	O4—Cu2—O19—Cl2	133.1 (5)
N2—C2—C3—Se	179.5 (2)	N6—Cu2—O19—Cl2	−56.3 (5)
C1—C2—C3—Se	−3.9 (5)	O15—Cu2—O19—Cl2	9.3 (12)
C12—Se—C3—C2	69.3 (3)		

### Hydrogen-bond geometry (Å, º)

**Table:**

*D*—H···*A*	*D*—H	H···*A*	*D*···*A*	*D*—H···*A*
O1—H2*O*1···O10	0.83 (2)	1.93 (2)	2.737 (3)	164 (4)
O1—H1*O*1···O18^i^	0.81 (2)	2.00 (2)	2.801 (6)	174 (5)
O1—H1*O*1···O20^i^	0.81 (2)	1.99 (3)	2.747 (6)	155 (4)
O2—H1*O*2···O13^ii^	0.83 (2)	1.85 (2)	2.660 (3)	163 (4)
O2—H2*O*2···O3^iii^	0.83 (2)	1.99 (2)	2.805 (4)	167 (4)
O3—H1*O*3···O11^i^	0.83 (2)	2.03 (2)	2.842 (4)	166 (4)
O3—H1*O*3···O12^i^	0.83 (2)	2.47 (3)	3.130 (4)	137 (4)
O4—H1*O*4···O10^iv^	0.83 (2)	1.94 (2)	2.762 (3)	174 (4)
O4—H2*O*4···O1*W*^v^	0.84 (2)	1.88 (2)	2.717 (4)	174 (5)
O5—H1*O*5···O13	0.82 (2)	1.87 (3)	2.617 (4)	150 (4)
O5—H2*O*5···O16	0.84 (2)	1.97 (3)	2.719 (11)	148 (4)
O5—H2*O*5···O16	0.84 (2)	1.97 (3)	2.719 (11)	148 (4)
O5—H2*O*5···O22	0.84 (2)	2.07 (3)	2.784 (10)	142 (4)
O1*W*—H2*W*1···O6^iv^	0.83 (2)	2.10 (3)	2.800 (4)	142 (4)
O1*W*—H1*W*1···O16^vi^	0.84 (2)	2.12 (3)	2.833 (9)	144 (4)
O1*W*—H1*W*1···O22^vi^	0.84 (2)	2.23 (3)	2.977 (11)	149 (4)
N2—H1*N*2···O11	0.86	1.98	2.829 (4)	168
N5—H1*N*5···O1*W*	0.86	1.94	2.762 (4)	160

Symmetry codes: (i) −*x*+1, −*y*+1, −*z*+1; (ii) *x*−1, *y*+1, *z*; (iii) −*x*, −*y*+1, −*z*+1; (iv) *x*, *y*−1, *z*; (v) −*x*+1, −*y*, −*z*+1; (vi) *x*−1, *y*, *z*.
